# Outcomes of tricuspid annuloplasty with and without prosthetic rings: a retrospective follow-up study

**DOI:** 10.1186/s13019-015-0281-2

**Published:** 2015-06-06

**Authors:** Wen-Jun Ren, Ben-Gui Zhang, Jia-Sheng Liu, Yong-Jun Qian, Ying-Qiang Guo

**Affiliations:** Department of Cardiovascular Surgery, West China Hospital, Sichuan University, Chengdu, Sichuan P.R. China

**Keywords:** Rheumatic heart disease, Tricuspid insufficiency, Tricuspid annuloplasty, Prosthetic ring, Quality of life

## Abstract

**Background:**

The efficacies of tricuspid valve repair, risk factors for treatment failure and postoperative quality of life have not been thoroughly evaluated in patients with tricuspid insufficiency associated with rheumatic heart disease (RHD). We therefore reviewed our experience with ring and non-ring tricuspid annuloplasty for the treatment of functional tricuspid insufficiency (TI) in RHD.

**Methods:**

This retrospective, follow-up study involved 74 RHD patients who underwent either non-ring annuloplasty (De Vega procedure; 34 patients, 45.95 %) or ring annuloplasty (40 patients, 54.05 %) along with concurrent mitral or/and aortic valve replacement. Operation time, cardiopulmonary bypass time, aortic clamping time, intensive care unit stay and extubation time were recorded. Echocardiographic findings and Short Form (SF)-36 scores were compared between the two groups.

**Results:**

In hospital mortality and complications were similar in the two study groups (P = 0.6755). At 1 week, 1 month, 6 months, 1 year, 2 years and even longer after the operation, the Kaplan–Meier curve of freedom from mild and above recurrent TI showed significantly better efficacy in the ring annuloplasty group than the De Vega procedure group (log rank P = 0.0377). Risk factors for recurrent TI included high pulmonary artery systolic pressure (PASP) and non-ring annuloplasty (PASP: hazard ratio = 1.52; non-ring: hazard ratio = 1.42). The mental component summary score at 1 year after the operation did not significantly differ between the two groups (P = 0.6446), but the physical component summary score was significantly better in the ring annuloplasty group (P = 0.0037).

**Conclusion:**

Compared with non-ring annuloplasty, ring annuloplasty was associated with improved survival, decreased TI recurrence and higher quality of life in RHD patients undergoing tricuspid valve repair combined with mitral and/or aortic valve replacement.

## Background

Despite the recent dramatic decline in rheumatic fever in developed countries, rheumatic heart disease (RHD) is still associated with significant morbidity and mortality in developing countries which accounts for more than 200,000 deaths annually [1]. Without treatment, the tricuspid valve insufficiency in RHD patients may worsen over time, leading to severe symptoms, advanced congestive heart failure and even death [2]. Tricuspid insufficiency (TI) secondary to left heart lesions such as chronic mitral or/and aortic valve disease, which causes pulmonary hypertension, right ventricular volume overload, is mostly functional in nature and was associated with annular dilation/remodeling. Therefore, correction of the left heart lesion and strengthening of the tricuspid annulus in this situation removes the impetus for progressive insufficiency [3].

Tricuspid valve repair is beneficial for treating severe TI in patients requiring mitral valve surgery, according to the 2006 guidelines of the American College of Cardiology/American Heart Association for the management of patients with valvular heart disease [4]. Recently, bicuspidization has since been superseded by other techniques that aim to remodel the annulus by maintaining a trileaflet valve with a more physiological and stable annulus [5]. “Non-ring” annuloplasties (such as the De Vega and Peri-Guard procedures) have the advantages of simplicity and low cost; however, some longitudinal studies have showed a higher risk of recurrent TI after the De Vega techniques than after ring annuloplasty [6]. Novel techniques of tricuspid valve repair include the use of flexible and rigid prosthetic rings or three-dimensional rings, and flexible prosthetic bands [7]. According to Tang et al., the advantages of rigid or flexible prosthetic rings may be related to the prevention of annular dilation, right ventricular volume overload and right ventricular failure [8]. Although studies on the repair of functional TI have shown potential advantages of ring annuloplasty [9], the efficacy of these techniques in rheumatic TI, the risk factors for treatment failure and the resultant quality of life have not yet been thoroughly evaluated.

The purpose of this study was therefore to review our experience with patients who had undergone different tricuspid valve repair procedures (De Vega procedure or ring annuloplasty) for the treatment of TI.

## Methods

### Patients

From March 2009 to September 2012, a total of 74 patients diagnosed with valve disease of rheumatic etiology, significant heart failure (New York Heart Association [NYHA] class, II–IV) and marked TI underwent tricuspid annuloplasty, which was performed by the same surgeon, in our institution. There were 11 male patients and 63 female patients, whose ages ranged from 33 to 66 years (mean, 48.4 years). All patients underwent preoperative echocardiography during the 3 months preceding the surgery. The De Vega procedure was performed in 34 (45.95 %) patients, and Cosgrove-Edwards flexible bands (Edwards Lifesciences, Irvine, California, United States) were used in 40 (54.05 %) patients. All the TIs were functional, so patients with tricuspid stenosis, leaflet calcification or severe leaflet thickening were excluded from our analysis. Preoperative, operative and postoperative data were collected into a professional database. All patients provided informed consent for inclusion in the study. The study protocol was approved by the ethics committee of our hospital.

Prior to the surgery, echocardiographic assessments were performed to grade the severity of TI according to color Doppler flow criteria [8], evaluate ventricular function and estimate pulmonary artery systolic pressure (PASP) on continuous Doppler scanning [10].

### Operative techniques

All tricuspid valve surgery was performed after concomitant cardiac procedures, including aortic and/or mitral valve surgery with median sternotomy and moderately hypothermic cardiopulmonary bypass (CPB). Tricuspid valve repair was performed under cardiac arrest. In the case of the De Vega technique, a 2–0 pledget-supported Ethibond mattress suture (Ethibond Excel 2–0 sutures, Johnson & Johnson Intl., USA) was placed at the junction of the annulus and right ventricular free wall, running from the anteroseptal commissure to the posteroseptal commissure [11]. This operation was performed in our hospital until June 2010. Since June 2010, we switched to ring annuloplasty, which employs multiple interrupted, pledgeted 2–0 Ethibond sutures placed at the atrioannular junction. After the flexible band was placed, the sutures were tied [12]. All prosthetic valves (left-sided valve replacement) were St. Jude Medical (SJM) mechanical prosthesis (St. Jude Medical, St. Paul. Mn, USA).

### Follow-up

All surviving patients were followed up at 1 week, 1 month, 1 year, 2 years and even longer after the surgery. All follow-up data were collected on a data collection sheet and entered into a professional database between March 2009 and March 2014. Postoperative echocardiogram reports were available for all patients (1 week, 1 month, 6 months, 1 year and 2 years after the operation). The mean follow-up was 2.83 years (range, 2–4 years). Follow up of 94.6 % was achieved. Follow-up was performed via telephone interviews and the collection of echocardiographic data.

The primary clinical end points of this study were defined as the all-cause mortality rate and operative-related or cardiac-related severe morbidity rates. The secondary clinical end point was recurrent TI on postoperative echocardiographic examination.

### Quality of life

The Short Form (SF)-36 Health Survey is one of the most extensive, standardized, self-administered, generic questionnaires produced within the framework of the International Quality of Life Assessment [13, 14]. The responses to each question on the survey were summed and transformed to give eight scores between 0 and 100, with higher scores indicating a better state. We collected the SF-36 questionnaires (in Chinese) before and at 1 year after the surgery [15].

### Statistical analysis

Categorical variables (Fisher exact test) were expressed as percentages, and continuous variables (Student *t*-test) were expressed as mean ± standard deviation. The Wilcoxon rank sum test was used for variables with non-parametric distribution. The rates of TI recurrence (including mild, moderate and severe degree TI) were compared using the Kaplan–Meier curve and log-rank test. Significance was assumed for P < 0.05. We performed mixed-model repeated-measures analysis and longitudinal ordinal logistic regression for each tricuspid annuloplasty technique. To determine the risk factors for postoperative TI recurrence, we performed multivariate Cox regression. All statistical analyses were performed using the Statistical Package for Social Sciences, version 17.0 (SPSS Inc., Chicago, IL, USA).

## Results

### Baseline characteristics

The preoperative and surgical characteristics of the patients are shown in Table 1. The two groups were well matched and similar with regard to all pretreatment characteristics. Furthermore, no significant difference was found between the groups in terms of age, NYHA class, left atrial size, left ventricular ejection fraction, PASP, and occurrence rate of TI.

**Table 1:** Characteristics of the patients

Characteristic	Annuloplasty without ring (De Vega procedure) (n = 34)		Annuloplasty with ring (n = 40)		*P* value
Age (yr ± SD)	47.2 ± 7.3		49.5 ± 8.7		0.241
Male	7	20.6 %	4	10.0 %	0.326
Preoperative NYHA ClassIII-IV	33	97.1 %	38	95.0 %	0.945
Preoperative TI grade					
None (n)	0	0.0 %	0	0.0 %	0.131
Mild (n)	8	23.5 %	14	35.0 %	
Moderate (n)	18	52.9 %	23	57.5 %	
Severe (n)	8	23.5 %	3	7.5 %	
Preoperative TS	0	0	0	0	
Preoperative mitral valve disease					
None (n)	0	0.0 %	0	0.0 %	0.808
Stenosis (n)	21	61.7 %	24	60.0 %	
Insufficiency (n)	2	5.8 %	4	10.0 %	
Stenosis and insufficiency (n)	11	32.4 %	12	30.0 %	
Preoperative aortic valve disease					
None (n)	17	50.0 %	21	52.5 %	0.98
Stenosis (n)	1	2.9 %	1	2.5 %	
Insufficiency (n)	7	20.6 %	9	22.5 %	
Stenosis and insufficiency (n)	9	26.5 %	9	22.5 %	
Preoperative					
LVEF	55.9 ± 9.1		58.1 ± 7.1		0.253
RVD	22.9 ± 5.1		21.9 ± 3.4		0.29
LVD	49.6 ± 6.66		47.9 ± 8.8		0.351
PASP	51.5 ± 16.6		49.9 ± 14.2		0.668
1 year Postoperative					
LVEF	61.9 ± 10.0		61.0 ± 8.8		0.733
RVD	20.7 ± 2.9		20.0 ± 2.2		0.277
LVD	44.5 ± 5.6		45.6 ± 4.1		0.37
PASP	36.5 ± 7.4		36.0 ± 8.3		0.903
Concomitant procedures					
MVR (n)	22	64.7 %	32	80.0 %	0.132
AVR (n)	0	0	0	0	
MVR + AVR (n)	12	35.3 %	8	20.0 %	

*AVR* aortic valve replacement, *LVEF* left ventricular ejection fraction, *LVD* left ventricular diameter, *RVD* right ventricular diameter, *MVR* mitral valve replacement, *NYHA* New York Heart Association, *PASP* pulmonary arterial systolic pressure, *SD* standard deviation, *TI* tricuspid valve insuffiency, *TS* tricuspid valve stenosis

### CPB, aortic clamping time and intensive care unit stay

The mean aortic clamping time in all patients was 88.0 ± 25.9 min. It was slightly, but not significantly, shorter in the ring annuloplasty group (86.9 ± 25.3 min) than in the De Vega procedure group (89.3 ± 26.8 min; P = 0.897). The mean CPB time was 125.7 ± 31.2 min in the ring annuloplasty group and 125.3 ± 31.0 min in De Vega procedure group (P = 0.701). In addition, the duration of intensive care unit stay and extubation time did not significantly differ between the two study groups (ICU stay P = 0.471, extubation time P = 0.610).

### Mortality, reoperations and complications

**Fig. 1:**
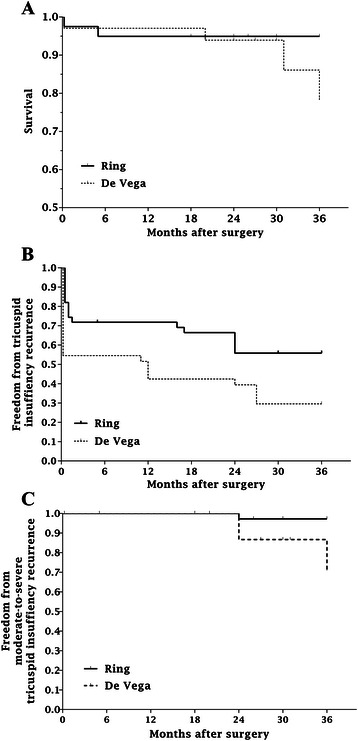
Kaplan–Meier curves after surgery. **a** Survival curves in the De Vega procedure group and the ring annuloplasty group. (log rank P = 0.6755) **b** Freedom from mild and above recurrence of tricuspid insufficiency in the De Vega procedure group and the ring annuloplasty group. (log rank P = 0.0377) **c** Freedom from moderate-to-severe recurrence of tricuspid insufficiency in the De Vega procedure group and the ring annuloplasty group (log rank P = 0.0970)

The 30-day mortality rate was 2.9 % (one patient) in the non-ring annuloplasty (De Vega procedure) group; the cause of death was sudden cardiac arrest. In addition, there were three late deaths: one cardiac failure and two severe bleeding associated with warfarin. In the ring annuloplasty group, one patient (2.5 %) died of cardiac rupture within 30 days after the surgery, and there was one late death that was caused by warfarin overdose. The Kaplan–Meier survival rates were 88.2 % at 2 years in the De Vega procedure group and 95.0 % in the ring annuloplasty group (Fig. 1a, log rank P = 0.6755). In two patients, severe postoperative complications occurred: reoperation due to surgical bleeding (one patient, ring annuloplasty group) and atrioventricular dissociation requiring pacemaker implantation (one patient, ring annuloplasty group). The rate of severe postoperative complications was slightly, but not significantly, greater in the ring annuloplasty group (5.8 %) than in the De Vega procedure group (0 %; P = 0.208).

### Evaluation of tricuspid annuloplasty

To determine the overall efficacy of the two annuloplasty techniques, we determined the TI grade at the last follow-up and assessed freedom from recurrence of more than mild degree and only moderate-to-severe degree TI by using the Kaplan–Meier survival curve (Fig. 1b, c) from 1 week to more than 2 years after the operation.

At 1 week after the operation, the overall TI recurrence (including Mild degree above) differed significantly between the two groups (45.5 % in the De Vega procedure group, 17.5 % in the ring annuloplasty group; P <0.01). But the moderate-to-severe TI recurrence was comparable among each group (2.9 % in the De Vega procedure group, 2.5 % in the ring annuloplasty group; P = 0.907), Meanwhile, decreased PASP and anteroposterior diameters of the right atrium and right ventricle were observed in both groups, but not significant (P > 0.05). At 1 month after the operation, TI recurrence (including Mild degree above) was also significantly higher in the De Vega procedure group (46.4 %) than in the ring annuloplasty group (23.5 %; P = 0.030). Meanwhile the moderate-to-severe TI recurrence was just equal to the data at 1 week after the operation (2.9 % in the De Vega procedure group, 2.5 % in the ring annuloplasty group; P = 0.907). The decrease in PASP and anteroposterior diameters of the right atrium and ventricle was similar in both groups (P > 0.05). At 1 year postoperatively, the rate of TI recurrence (including mild degree or above) was still significantly greater in the De Vega procedure group (58.0 %) than in the ring annuloplasty group (27.5 %; P < 0.001), and the rate of moderate-to-severe TI recurrence was significantly more common in the De Vega procedure group (17.6 %) than in the ring annuloplasty group (2.5 %; P = 0.043). None of the patients had severe TI at this time. At 2 years postoperatively, among all the alive patients, mild, moderate and severe TI were present in 59.3 %, 18.0 % and 0 % patients, respectively, in the De Vega procedure group and 42.3 %, 2.7 % and 0 % patients, respectively, in the ring annuloplasty group. The rate of moderate-to-severe TI recurrence was still significantly more common in the De Vega procedure group (18.0 %) than in the ring annuloplasty group (2.7 %) Both in terms of efficacy and duration, ring annuloplasty surpassed the De Vega procedure for the treatment of functional TI associated with RHD.

### Risk factors for TI recurrence

The following variables were entered in the multivariate Cox regression model to identify significant independent predictors of survival and event-free survival: type of tricuspid valve repair surgery, age, gender, preoperative NYHA class, preoperative PASP, preoperative right ventricular dysfunction, preoperative TI, tricuspid valve repair type, preoperative mitral insufficiency or/and stenosis, preoperative left ventricular ejection fraction, concomitant mitral valve surgery and concomitant aortic valve surgery. According to the Cox model, annuloplasty type (De Vega procedure) and high preoperative PASP were risk factors for TI recurrence (including mild to severe TI; Table 2). Low left ventricular ejection fraction was also a risk factor for TI recurrence. In patients with better left heart function, preoperative TI may indicate a greater degree of valve dysfunction, such as annular dilation or valve tethering, which may hamper tricuspid valve repair. Preoperative and follow-up NYHA classes were not found to be significant factors. Similarly, right ventricular dysfunction (high right ventricular diameter and body edema) was not a risk factor for TI recurrence.

**Table 2:** Multivariate regression analysis of the risk factors for TI recurrence

Variable	*B* value	*P* value	Hazard ratio	SE
Preoperative PASP	0.51	0.018	1.52	0.22
Non-ring Annuloplasty, (De Vega procedure)	0.46	0.051	1.47	0.24

*PASP* pulmonary arterial systolic pressure, *SE* standard error, *TI* tricuspid insufficiency

### Quality of life assessment

The preoperative parameters on the SF-36 scale did not significantly differ between the two groups (physical functioning, P = 0.917; role-physical, P = 0.555; bodily pain, P = 0.216; general health, P = 0.592; vitality, P = 0.416; social functioning, P = 0.085; role-emotional, P = 0.332; mental health, P = 0.142). At the 1-year follow-up, the mean physical component summary (PCS) score was significantly higher in the ring annuloplasty group (52.58 ± 1.258) than in the De Vega procedure group (47.40 ± 1.163; P = 0.0037). The mean mental component summary (MCS) score did not significantly differ between the De Vega procedure group (49.64 ± 1.048) and the ring annuloplasty group (48.98 ± 0.9682; P = 0.6446).

The comparison of the pre- and postoperative data between the two study groups has been shown in Table 3. Subgroup analysis revealed that patients in the De Vega procedure group experienced significant improvement in five of the eight health domains on the SF-36: physical functioning, role-physical, general health, social functioning and role-emotional. Meanwhile, the ring annuloplasty group patients experienced significant improvement in all health domains, except for bodily pain (P = 0.204).

**Table 3:** Comparison of pre-and postoperative SF-36 scores between the ring and non-ring annuloplasty groups

Domain and summary scores		De Vega procedure	Ring annuloplasty
Physical Functioning	Pre	33.60 ± 10.86	34.67 ± 15.31
	Post	46.11 ± 13.84	52.67 ± 11.04
	*P* Value	**<0.001**	**<0.001**
Role-physical Problem	Pre	30.34 ± 15.38	32.92 ± 13.00
	Post	49.65 ± 14.17	53.75 ± 14.37
	*P* Value	**<0.001**	**<0.001**
Body Pain	Pre	47.46 ± 16.04	47.78 ± 14.00
	Post	51.16 ± 15.32	52.22 ± 12.75
	*P* Value	**0.320**	**0.204**
General Health Perception	Pre	39.31 ± 9.19	38.17 ± 15.95
	Post	46.39 ± 13.29	51.67 ± 11.32
	*P* Value	**<0.001**	**<0.001**
Vitality	Pre	42.17 ± 9.16	43.89 ± 11.28
	Post	46.83 ± 10.87	49.17 ± 9.90
	*P* Value	**0.077**	**0.038**
Social Functioning	Pre	35.67 ± 9.44	37.50 ± 9.52
	Post	46.17 ± 9.35	47.08 ± 9.66
	*P* Value	**<0.001**	**<0.001**
Role-emotional Problem	Pre	45.18 ± 9.65	43.51 ± 14.64
	Post	57.4 ± 13.71	53.70 ± 11.42
	*P* Value	**<0.001**	**0.002**
Mental Health	Pre	47.00 ± 14.36	37.78 ± 12.16
	Post	48.17 ± 13.29	45.97 ± 14.48
	*P* Value	**0.745**	**0.011**
PCS	Pre	38.61 ± 6.55	38.38 ± 8.34
	Post	47.40 ± 6.97	52.58 ± 6.89
	*P* Value	**<0.001**	**<0.001**
MCS	Pre	42.50 ± 5.69	40.67 ± 5.73
	Post	49.64 ± 5.74	48.98 ± 5.81
	*P* Value	**<0.001**	**<0.001**

*MCS* mental component summary, *PCS* physical component summary. Bold face numbers shows the *P* < 0.05

## Discussion

Most studies of tricuspid valve repair have focused on the indications for intervention, the technique of surgery, survival rates and reoperation rates [10, 16]. Few studies have evaluated the clinical outcomes and quality of life after tricuspid valve annuloplasty in RHD patients [17]. Functional TI with concomitant left-sided lesions, such as mitral or aortic valve disease in RHD, is associated with high mortality and increased risk of adverse events. In the western region of China, TI associated with RHD often occurs in younger patients (in our study, the mean age was 48.4 years) than does TI related to other causes. Our study has shown that although the use of a prosthetic ring did not significantly decrease the incidence of adverse clinical outcomes and mid-term mortality, it effectively alleviated TI recurrence and improved the quality of life in patients with rheumatic mitral and/or aortic valve disease.

Bernal et al. believed that annuloplasty rings were more effective than the De Vega procedure in preventing late TI after mitral valve repair for RHD [6]. Our study also demonstrated that ring annuloplasty had more efficacies in restoring and maintaining tricuspid valve function immediate after surgery, this result also duplicated in 1 month, 1 year and 2 years follow-up which indicating annuloplasty is a more durable method for TI repair. Different from patient with degenerated heart valve disease, left-sided valve of RHD is often too difficult to repair due to marked thickening and calcification of the valve tissue. As show in our study, all the patients received prosthetic mitral valve replacement rather than repair, which could completely change the native ring structure and subsequently to some extend affect the geometric shape of native tricuspid annulus. In this condition, rather than constricting of the dilated tricuspid annulus, better maintaining its geometric shape through an annuloplasty ring may be of paramount importance to ensure a durable repair effect. Also, in rheumatic mitral valve disease, left atrial pressure and PASP increase over time in nearly all patients; this leads to pressure overload of the right ventricle and induces right ventricular enlargement [18]. If the PASP is markedly increased, or the annulus moderately dilated, relatively minor leaflet disease may lead to functional TI [19]. RHD patients tend to have poor right heart function even before the occurrence of functional TI; therefore, the correction of even mild or moderate TI in these patients necessitates a more stable and durable annuloplasty method. Thus, the prosthetic ring technique may be selected as primary choose for the correction of functional TI in RHD patients. In our study, the multivariate Cox regression model revealed that high preoperative PASP and non-ring annuloplasty had a negative impact on rate of freedom from TI recurrence.

SF-36 scale is a validated measure of the overall physical and mental health status, though it does not contain disease-specific questions [20]. To our best knowledge, our study is the first to provide information on changes in the quality of life of patients after tricuspid valve repair surgery [21, 22]. An important goal of interventions is to provide a subjective sense of satisfaction with the treatment. The results of our study demonstrate that quality of life was significantly improved 1 year after the surgery as for both group. However, according to the PCS score, the extent of postoperative physical improvement in health significantly differed between the two groups. Meanwhile, compared with the De Vega procedure, the ring annuloplasty group patients experienced more improvement in almost all health domains of SF-36 scale. These results suggest that the fewer TI recurrence may cause a better symptom, such as fewer edema or hepatic congestion after the surgery, which affect the subjective sense of patients [19]. These trends require further research, particularly, with regard to their effects on long-term clinical outcomes.

The limitations of this study include its single-center design and retrospective nature, with all of the inherent limitations of such investigations. A non-randomized study with short duration and small sample size may generate potential bias. Moreover, we did not record and analyze the diameter of the tricuspid annulus because this data was not routinely recorded in our hospital. Nevertheless, our results may help cardiac surgeons select an appropriate repair technique for the treatment of tricuspid insufficiency due to rheumatic heart valve disease.

## Conclusion

Ring annuloplasty was more effective than the De Vega procedure in treating functional TI in RHD patients undergoing tricuspid valve repair combined with mitral and/or aortic valve replacement. Our study suggests that tricuspid valve repair surgery, especially ring annuloplasty, significantly improved the quality of life of the patients. These findings indicate that prosthetic ring annuloplasty for the correction of functional TI in RHD may provide a greater improvement in mid-term clinical outcomes and quality of life than non-ring annuloplasty (De Vega procedure).

### Consent

Note: Written informed consent was obtained from all the patients. A copy of the written consent is available for review by the Editor-in-Chief of this journal.

## Abbreviations

CPB: Cardiopulmonary bypass
MCS: Mental component summary
NYHA: New York Heart Association
PASP: Pulmonary artery systolic pressure
PCS: Physical component summary
RHD: Rheumatic heart disease
SF-36: Short Form-36
TI: Tricuspid insufficiency
